# Biodegradable PBAT films with in situ synthesized tannic acid/ZnS nanohybrids for active packaging, offering antioxidant, antibacterial, and UV-shielding properties

**DOI:** 10.1038/s41598-025-30768-x

**Published:** 2025-12-11

**Authors:** Maryam Niksefat, Reza Bagheri, Ali Pourjavadi

**Affiliations:** 1 Institute for Nanoscience and Nanotechnology, Sharif University of Technology, Tehran, Iran; 2 Department of Materials Science and Engineering, Sharif University of Technology, P.O. Box 11155-9466, Tehran, Iran; 3 Department of Chemistry, Sharif University of Technology, P.O. Box 11365-9516, Tehran, Iran

**Keywords:** Biopolymer nanocomposite, Organic–inorganic hybrid, Antimicrobial activity, UV protection, Water vapor barrier, Food packaging, Green synthesis, Chemistry, Materials science, Nanoscience and technology

## Abstract

Active food packaging that provides antioxidant and antimicrobial protection can extend product shelf life, but adding such features to polymers often affects material performance. In this study, we developed a biodegradable poly(butylene adipate-co-terephthalate) (PBAT) film incorporating in situ synthesized tannic acid (TA)/zinc sulfide (ZnS) hybrid nanostructures to add antioxidant, antibacterial, and UV-shielding functions. The TA/ZnS nanohybrids were created using a green in situ co-precipitation method and evenly dispersed in the PBAT matrix to form nanocomposite films. The resulting films showed strong bioactivity, achieving 90.46% DPPH free radical scavenging and inhibition zones over 3.0 cm against both *E. coli* and *S. aureus*. They also offered about 97% UV blocking and improved moisture barrier properties, with a 40.9% decrease in water vapor permeability and a 13% increase in water contact angle (surface hydrophobicity). However, these improvements came with roughly a 30% reduction in tensile strength, highlighting a trade-off between added functionality and mechanical performance. Importantly, the composite kept enough flexibility and strength for practical applications. In conclusion, the organic–inorganic synergy between tannic acid and ZnS created a bioactive, UV-protective film that can help extend food shelf life while remaining biodegradable. This green, scalable method shows potential for sustainable next-generation active packaging.

## Introduction

Organic–inorganic hybrid nanostructures have emerged as a promising class of materials that combine the mechanical and thermal stability of inorganic components with the flexibility, versatility, and bioactivity of organic molecules^1,2^. This synergy has enabled the development of advanced materials for applications such as food packaging, where the demand for smart, sustainable, and multifunctional solutions is steadily increasing^3–5^ .In particular, nanocomposite films derived from biodegradable polymers offer functionalities beyond conventional passive barriers—such as antibacterial, antioxidant, UV-shielding, and moisture-controlling properties—while minimizing environmental impact^4–6^.

Among naturally derived functional molecules, tannic acid (TA) stands out due to its polyphenolic structure, which contains multiple hydroxyl groups capable of scavenging free radicals, chelating metal ions, and disrupting microbial membranes^7–9^. These chemical features give TA both antioxidant and antimicrobial activity, which are critical for preserving food quality and extending shelf life^10–12^. In addition, TA can provide UV-blocking and thermal stabilization when incorporated into polymer films^13^. However, free TA in polymer matrices is prone to migration and leaching, which reduces its long-term effectiveness in packaging systems^12^.

On the other hand, zinc sulfide (ZnS) nanoparticles offer complementary properties. As a wide-bandgap semiconductor (~ 3.6 eV), ZnS exhibits excellent UV absorption and photostability, while maintaining transparency in the visible light range, making it an attractive option for consumer-facing packaging applications^14,15^. Furthermore, under UV light, ZnS can produce reactive oxygen species (ROS) that contribute to antimicrobial activity^16,17^. Compared to more common fillers such as ZnO or TiO₂, ZnS exhibits reduced photocatalytic activity in the visible range, which protects the underlying polymer matrix^18^. Despite these advantages, ZnS nanoparticles tend to aggregate and disperse poorly in polymers, requiring surface modification or hybridization strategies^17,19^.

**Fig. 1:**
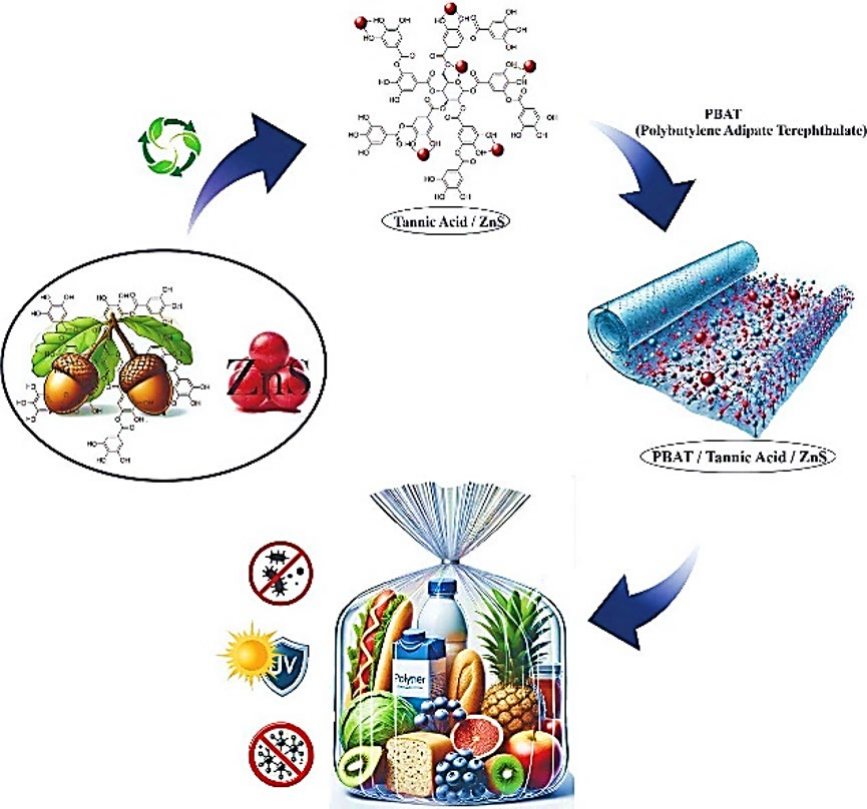
Schematic illustration of the synthesis route and multifunctional roles of the PBAT/TA-ZnS film for active food packaging.

To overcome these limitations and combine the functionalities of TA and ZnS, we developed an in situ synthesized TA/ZnS hybrid nanostructure using a green precipitation method. We incorporated it into a biodegradable PBAT (poly (butylene adipate-co-terephthalate)) matrix. The in situ approach enhances interfacial interactions and ensures a homogeneous distribution of the hybrid particles, thereby avoiding the phase separation and aggregation issues commonly encountered in ex-situ blending methods^19,20^. Recently, PBAT-based nanocomposites have gained considerable attention as promising candidates for sustainable food packaging owing to their biodegradability, flexibility, and tunable physical properties. Several recent studies have demonstrated that the incorporation of inorganic nanofillers or bioactive compounds into PBAT can significantly improve its UV-shielding, antibacterial, and barrier performances while maintaining its mechanical integrity. For example, hybrid systems such as PBAT/TiO₂, g-C₃N₄/TiO₂, and tannic acid–gallic acid crosslinked chitosan blends have been shown to extend the shelf life of packaged fruits and vegetables by enhancing light protection and antimicrobial efficiency^21–23^. These advances confirm the growing potential of PBAT nanocomposites for next-generation active and biodegradable food packaging materials. PBAT, as a compostable aliphatic-aromatic copolyester, provides good flexibility and mechanical properties, making it an ideal host for food packaging films^16,24,25^. Several recent studies have reported the incorporation of TA into ZnO or other polymer matrices for multifunctional performance^26–28^; however, to the best of our knowledge, this is the first report of an in situ synthesized TA/ZnS hybrid embedded in a PBAT matrix for active packaging. This study aims to synthesize and characterize the hybrid nanostructure, fabricate nanocomposite films, and evaluate their antioxidant, antibacterial, UV-blocking, and water barrier properties, with a focus on scalability and sustainability for food protection applications^29–31^. A schematic representation of the synthesis strategy and packaging functionality of the developed nanocomposite is presented in Fig. 1.

## Materials and methods

### Materials

All reagents used in this study were of analytical grade and used as received without further purification. Tannic acid (TA), zinc chloride (ZnCl₂), and sodium sulfide nonahydrate (Na₂S·9 H₂O) were purchased from standard chemical suppliers. Poly(butylene adipate-co-terephthalate) (PBAT, grade ECOPOND^®^ A400) was supplied by Zhuhai Kingfa Biomaterial Co., Ltd. (Guangdong, China) and used as the biodegradable polymer matrix in pellet form. Chloroform was used as the primary solvent for film casting. Ethanol and deionized water were employed as solvents during synthesis and washing steps. Methanol and 2,2-diphenyl-1-picrylhydrazyl (DPPH) were used in the antioxidant activity assay.

### Synthesis of tannic Acid/Zinc sulfide nanostructures (TA/ZnS)

TA/ZnS hybrid nanostructures were synthesized using a green, aqueous in situ co-precipitation method under an inert argon atmosphere. Tannic acid (0.03 g) was first dissolved in 25 mL of deionized water and stirred for 1 h to activate its polyphenol groups. Zinc acetate dihydrate (1 mmol) was then added, allowing chelation between Zn²⁺ ions and the hydroxyl groups of TA. Thioacetamide (1.6 mmol) was introduced as a slow-release sulfur donor to promote controlled ZnS nucleation, followed by dropwise addition of 1 M NaOH (15 mL) to adjust pH and initiate precipitation. The reaction was carried out under mildly alkaline conditions (approximately pH 9–10) to facilitate thioacetamide hydrolysis and ZnS precipitation. The mixture was stirred for another hour, and the resulting nanohybrids were isolated by centrifugation, washed thoroughly with hot ethanol and water, and dried at 60 °C for 48 h.

### Preparation of nanocomposite films

Nanocomposite films were fabricated using PBAT as the polymer matrix and a solvent casting approach. PBAT (4 g) was dissolved in 50 mL of chloroform under vigorous magnetic stirring for 1 h to form a uniform solution. In parallel, TA/ZnS nanostructures (0.08 g, corresponding to 2 wt% relative to the polymer) were dispersed in 10 mL of chloroform using a Reliance Digital 5 L ultrasonic bath (Medisafe UK Ltd, Bishops Stortford, Hertfordshire, UK) operating at a frequency of 40 kHz and an output power of approximately 150 W for 30 min to ensure homogeneous dispersion of the nanostructures in the solvent. The nanofiller suspension was then slowly added to the PBAT solution and stirred vigorously for another hour, followed by a second ultrasonication step lasting 30 min to improve dispersion and prevent aggregation. The final mixture was cast onto levelled glass plates (12 × 8 cm) and dried in an oven at 60 °C to evaporate the solvent. The obtained films were peeled off and conditioned at 25 °C and 50% relative humidity for at least 72 h prior to characterization. Control films containing neat PBAT, PBAT with tannic acid (PBAT/TA), and PBAT with ZnS (PBAT/ZnS) were prepared following the same procedure, with 2 wt% additive loading in each case.

### Nanostructure characterization

#### Fourier transform infrared spectroscopy (FT-IR)

FT-IR spectroscopy was carried out using a Perkin Elmer Spectrum 2 spectrometer in the range of 4000–400 cm⁻¹ with a spectral resolution of 4 cm⁻¹. The spectra were used to confirm the chemical interactions between tannic acid and zinc sulfide by identifying characteristic functional groups. The nanocomposite was analysed as a powder, while the films were analysed in their film form. Peaks corresponding to hydroxyl groups (O-H), carbonyl groups (C = O), and Zn-S stretching vibrations were examined to ensure successful incorporation.

#### X-Ray diffraction (XRD)

The crystalline structure of the nanostructures was analysed using a PANalytical XPert Pro MPD diffractometer. Diffraction patterns were recorded in the 2θ range of 0.8°–156°, and crystallite sizes were calculated using the Scherrer equation. For film characterization, 1 × 1 cm film sections were used to determine the effect of nanostructure incorporation on crystallinity.

#### Thermogravimetric analysis (TGA)

Thermal stability was evaluated using a Linseis STA PT1600 analyser. Samples were heated in an atmospheric environment up to 450 °C and in a nitrogen atmosphere up to 750 °C to study weight loss profiles and thermal behavior.

#### Field emission scanning electron microscopy (FE-SEM)

Morphological analysis was performed using a ZEISS Sigma FE-SEM. Before analysis, samples were coated with gold to improve conductivity. Surface morphology, particle size, and dispersion of nanostructures were examined to assess homogeneity.

### Film characterization

####  Mechanical properties 

Mechanical properties, including tensile strength (TS), elastic modulus (EM), and elongation at break (EB), were measured according to the ASTM D882-88 standard using a universal testing machine7. Dumbbell-shaped samples with a precise length of 7 cm and an initial grip distance of 3 cm were prepared using standard molds. Film thickness was measured prior to testing using a digital micrometre with a precision of 1 μm, and the average thickness was calculated from five random measurements across each film. The tensile tests were conducted at a crosshead speed of 10 mm/min. Results were averaged over five replicates to ensure accuracy and reliability.

#### Water vapor permeability (WVP)

WVP was determined using the ASTM E95-96 standard gravimetric method. Films with dimensions of 75 × 75 mm were mounted in test cups containing 18 mL distilled water (cup depth: 25 mm, diameter: 68 mm). The cups were sealed to allow water evaporation exclusively through the films. Weight loss was measured at 25 °C and 50% relative humidity at hourly intervals for 8 h. The WVP was calculated using the equation:

$${\rm WVP = \frac{\Delta W \times L}{t \times A\: \Delta P}}$$

where ∆W is the weight difference (g), L is the film thickness (mm), t is time (s), A is the surface area (m²), and ∆P is the vapor pressure difference across the film31.

#### Water contact angle (WCA)

The water contact angle (WCA) of the films was determined using the sessile drop method. A 10 µL droplet of distilled water was carefully placed on the film surface, and high-resolution images of the droplet were captured using a smartphone camera positioned perpendicular to the sample surface. The contact angles were analysed using ImageMeter software (v3.5.2, https://imagemeter.com) to determine the angle accurately. Each measurement was repeated five times for every sample, and the reported values represent the average of these replicates. The films (30 × 100 mm) were fixed on a Teflon-coated steel base during measurement to ensure stability and precision.

#### UV-Vis spectroscopy and optical properties

The optical properties of the films, including UV-blocking and transparency, were evaluated using a Perkin Elmer LQS-ID-006 spectrometer. Light transmittance was measured in the UV region (280–400 nm) to determine UV-blocking capability and at 660 nm to assess transparency. Higher UV-blocking efficiency indicates improved protective performance, while lower light transmittance in the visible region reflects reduced film transparency.

#### Antibacterial properties

Antibacterial properties of PBAT-based films were evaluated using the agar diffusion method by determining the inhibition zone against Escherichia coli (Gram-negative) and Staphylococcus aureus (Gram-positive) strains. A 24-hour bacterial culture was first prepared in nutrient broth, and the optical density (OD) of the bacterial suspension was adjusted to 0.1 at 600 nm using a UV-Vis spectrophotometer, corresponding to a concentration of 10⁷ CFU/mL. A 0.1 mL aliquot of the bacterial suspension was spread evenly on nutrient agar plates using a sterile spreader. Each film sample was placed at the center of the plate, and the plates were incubated at 37 °C for 24 h. The inhibition zone was measured to evaluate antibacterial performance, and results were reported in millimeters.

### Thermal and antioxidant analyses (common for nanostructures and films)

#### Thermal analysis (TGA/DSC)

The thermal behaviour of both the TA/ZnS nanostructures and PBAT-based nanocomposite films was analysed using a Linseis STA PT1600 analyser. Thermogravimetric analysis (TGA) was performed to evaluate the thermal stability and weight loss profiles of the samples. The measurements were conducted under a nitrogen atmosphere from room temperature up to 750 °C at a heating rate of 10 °C/min. Differential scanning calorimetry (DSC) was also performed using the same instrument to study the thermal transitions of the films. Samples were heated from 25 °C to 200 °C at a rate of 10 °C/min under a nitrogen atmosphere to examine melting and crystallization behaviour.

#### Antioxidant properties

The antioxidant activity of both the TA/ZnS nanostructures and PBAT-based nanocomposite films was evaluated using the DPPH radical scavenging assay^7^. A 4 mg DPPH solution was prepared in 100 mL of methanol. For each sample type (nanostructures and films), 50 mg of material was added to 10 mL of the DPPH solution and incubated in the dark at room temperature for 30, 60, 120, and 180 min. The absorbance of the resulting solutions was measured at 517 nm using a UV–Vis spectrophotometer.

The radical scavenging activity (%) was calculated according to the following equation:

$$\% {\rm DPPH scavenging = \:\frac{(\text{A}\text{c}\:-\:\text{A}\text{s})}{\text{A}\text{c}} \times 100}$$

where Ac ​ and As ​ represent the absorbances of the control and the sample, respectively30.

#### Statistical analysis

All experiments were performed in triplicate, and the results are presented as mean ± standard deviation (*n* = 3). Statistical analysis was carried out using Microsoft Excel (v.2211, https://www.microsoft.com/excel) and OriginPro 2023 (v10.0.5.157, https://www.originlab.com/2023). A one-way ANOVA test (*p* < 0.05) was used to evaluate differences among samples. The results showed no statistically significant differences (*p* > 0.05) in tensile strength, elastic modulus, or elongation at break, confirming that the incorporation of TA, ZnS, and TA/ZnS did not alter the mechanical integrity of the PBAT matrix.

## Results and discussion

### Nanostructure characterization

The successful synthesis and detailed characterization of TA/ZnS nanostructures were confirmed using multiple complementary techniques, establishing a robust foundation for their multifunctional properties in active food packaging applications.

**Fig. 2:**
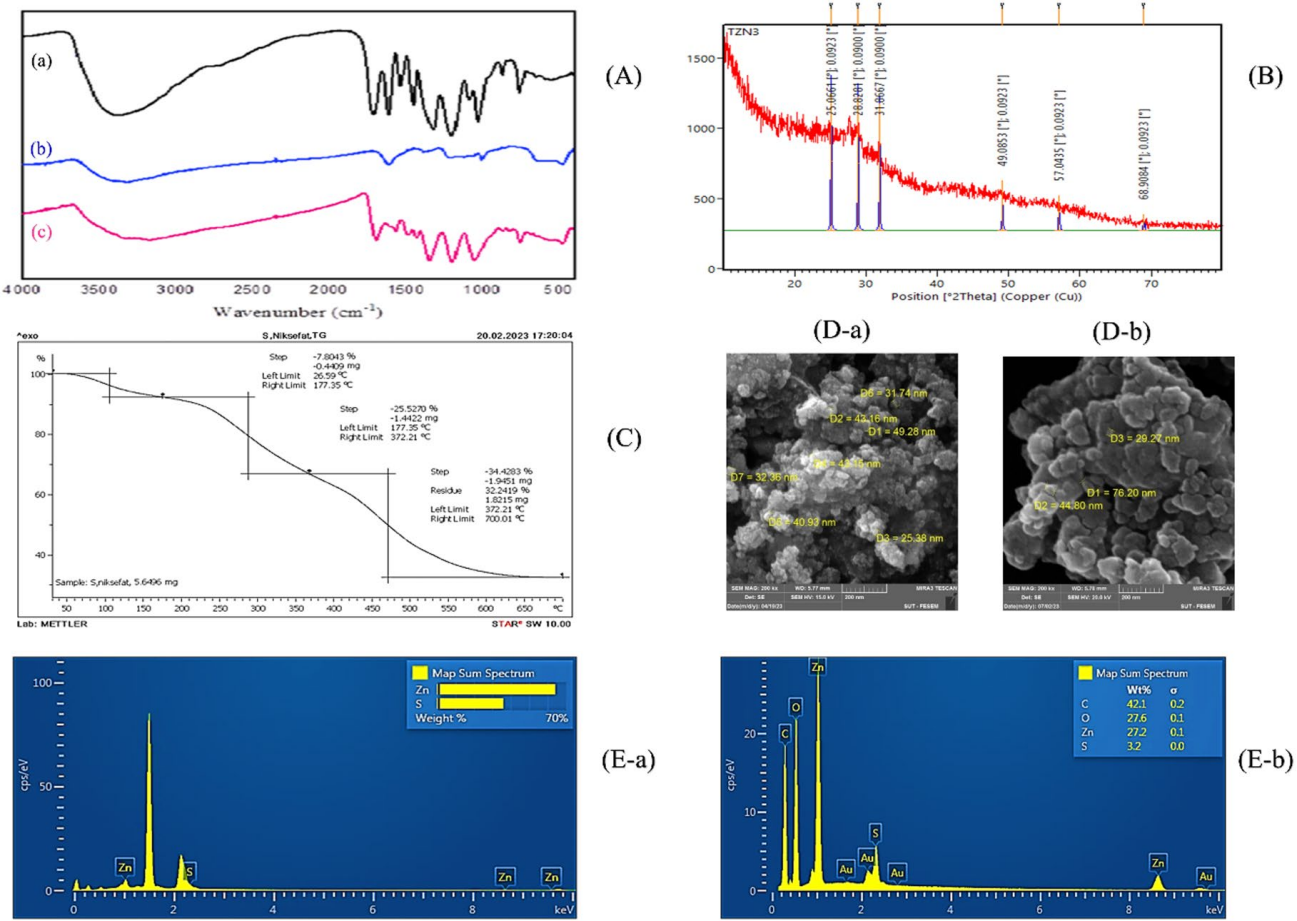
(**A**) FT-IR spectra of nanostructures: (a) Tannic acid, (b) ZnS, and (c) TA/ZnS. (**B**) XRD pattern of TA/ZnS nanostructure. (**C**) TGA curve of TA/ZnS nanostructure. (**D**) FE-SEM micrographs: (a) ZnS and (b) TA/ZnS. (**E**) EDX spectra: (a) ZnS and (b) TA/ZnS.

The successful synthesis of TA/ZnS hybrid nanostructures was confirmed through **Fourier-transform infrared (FT-IR)** spectroscopy, which provided insight into the chemical interactions between tannic acid and zinc sulfide. The FT-IR spectra of tannic acid, ZnS, and TA/ZnS hybrid nanostructures are presented together in Fig. 2(A) for comparison. The FT-IR spectrum of pure tannic acid displayed a broad absorption band centered around 3300 cm⁻¹, corresponding to the O–H stretching vibrations of phenolic hydroxyl groups responsible for its antioxidant functionality. Additional bands were observed at 1700 cm⁻¹ (C = O stretching of carbonyl groups), 1600–1450 cm⁻¹ (aromatic C = C vibrations), and 1000–1200 cm⁻¹ (C–O stretching), all of which are characteristic of the polyphenolic structure of tannic acid^32,33^.

In the ZnS spectrum (Fig. 2(A-b)), a distinct absorption peak appeared near 620 cm⁻¹, attributed to the Zn–S stretching vibration, confirming the successful formation of zinc sulfide^34^.

The FT-IR spectrum of the hybrid TA/ZnS nanostructures (Fig. 2(A-c)) exhibited characteristic peaks of both tannic acid and ZnS, including the broad O–H stretching band (~ 3300 cm⁻¹), the C = O and C–O vibrations (1700–1100 cm⁻¹), and the Zn–S stretching at 620 cm⁻¹ ^35^. The simultaneous presence of these bands indicates that tannic acid molecules were successfully incorporated onto the ZnS surface without significant chemical degradation. A slight red-shift of the O–H stretching band (from ~ 3330 to ~ 3290 cm⁻¹) and the C = O stretching band (from ~ 1700 to ~ 1690 cm⁻¹), accompanied by broadening in the 1000–1200 cm⁻¹ region, indicates the formation of hydrogen bonding and coordination interactions between the phenolic hydroxyl groups of tannic acid and Zn²⁺ ions in ZnS. These spectral changes confirm that tannic acid molecules are chemically associated with the ZnS surface through O–H···Zn and C = O···Zn interactions, while retaining their polyphenolic functionality essential for antioxidant activity^36,37^.

These spectral findings collectively confirm the formation of stable TA/ZnS hybrid nanostructures, in which tannic acid maintains its functional hydroxyl and carbonyl groups, critical for antioxidant and antimicrobial activity, while being chemically associated with ZnS nanoparticles.

**XRD analysis** (Fig. 2(B)) revealed the successful incorporation of ZnS nanocrystals within the nanocomposite film. Sharp diffraction peaks were observed at 2θ = 28.8°, 31.8°, 49.0°, 57.3°, and 68.9°, corresponding to the (111), (200), (220), (311), and (400) planes of cubic ZnS, which are consistent with the standard diffraction pattern of zinc sulfide (JCPDS card no. 05-0566)^38^. A broad peak centered around 2θ ≈ 25° was also detected, which can be attributed to the amorphous nature of tannic acid. Although the XRD pattern of pure tannic acid was not measured in this work, similar amorphous halos near 25° have been reported in previous studies^6^, confirming that the broad feature arises from the tannic acid matrix. Therefore, the TA/ZnS hybrid structure combines the crystalline ZnS phase with an amorphous polyphenolic matrix.

Using the Scherrer equation and a FWHM value of 0.00923 radians, the crystallite sizes of ZnS were estimated to range between 15.3 and 17.5 nm, with an average particle size of approximately 16 nm ^39^. The crystallite size (D) was calculated using the Debye–Scherrer equation:

$$\:D=\frac{K{\uplambda\:}}{{\upbeta\:}\:\text{c}\text{o}\text{s}{\uptheta\:}}$$

where D is the average crystallite size, K is the shape factor (typically 0.9), λ is the X-ray wavelength (0.154 nm for Cu Kα), β is the FWHM in radians, and θ is the Bragg angle.

The presence of an amorphous halo from TA alongside the crystalline ZnS reflections suggests a stable hybrid formation, where the organic TA is well-dispersed without disrupting the ZnS lattice. This structural compatibility likely plays a role in enhancing the antioxidant and antimicrobial functionalities of the nanocomposite films^40^.

**Thermogravimetric analysis (TGA)** of the TA/ZnS nanostructures (Fig. 2(C)) provided insight into their thermal decomposition profile and compositional stability. The TGA curve displayed three distinct weight-loss stages. The first stage, accounting for approximately 7.8% weight loss below 150 °C, is attributed to the evaporation of adsorbed moisture. The second stage, occurring between 150 and 372 °C with a weight loss of 25.5%, corresponds to the thermal degradation of the organic tannic acid component^41^.The third and most significant weight loss (34.4%) was observed from 372 °C to 700 °C, associated with the combustion of remaining carbonaceous residues.

The final residue of about 32.8% at 700 °C indicates the presence of thermally stable inorganic ZnS, which does not decompose in this temperature range^42^. These results confirm the successful hybridization of organic and inorganic phases and highlight the enhanced thermal stability of the material. Such stability is especially valuable in food packaging applications, where materials must withstand diverse temperature fluctuations during storage and transport without compromising structural or functional integrity.

**Morphological analysis via field-emission scanning electron microscopy (FE-SEM)** (Fig. 2(D)) confirmed the nanoscale structure of the synthesized TA/ZnS hybrid. The micrographs revealed well-dispersed, nearly spherical nanoparticles with apparent diameters in the range of 25–50 nm, as estimated using Digimizer software (v5.4.6, https://www.digimizer.com). These values are slightly larger than the crystallite sizes calculated from XRD data using the Scherrer Eq. (15.3–17.5 nm), which is attributed to the aggregation of several crystallites into single nanoparticles^42^.

The FE-SEM micrographs revealed quasi-spherical TA/ZnS nanoparticles that were homogeneously distributed without visible aggregation. These observations indicate that tannic acid acted as a stabilizing and capping agent during the synthesis, preventing uncontrolled particle growth^43^. This controlled morphology increases the surface area of the ZnS phase, which in turn enhances its interaction with reactive oxygen species (ROS) and contributes to the improved antioxidant and antimicrobial properties observed in the nanocomposite system^40^.

**Elemental composition analysis** of the TA/ZnS nanostructures was carried out using energy-dispersive X-ray spectroscopy (EDX), as presented in Fig. 2(E). The EDX spectrum revealed strong signals for zinc (Zn) and sulfur (S), confirming the presence of ZnS nanoparticles. In addition, notable peaks for carbon (C) and oxygen (O) were also observed, originating from the tannic acid component^44^.

Quantitative EDX analysis showed a Zn content of 27.2 wt% and S content of 3.2 wt%, with substantial contributions from carbon (42.1 wt%) and oxygen (27.6 wt%). The Zn/S atomic ratio closely matched the expected stoichiometry of ZnS^45^, supporting the successful formation of the hybrid structure. The carbon and oxygen signals further validated the incorporation of tannic acid into the nanostructure matrix, likely contributing to the improved surface interaction and stability observed in subsequent analyses^46^.

**Fig. 3:**
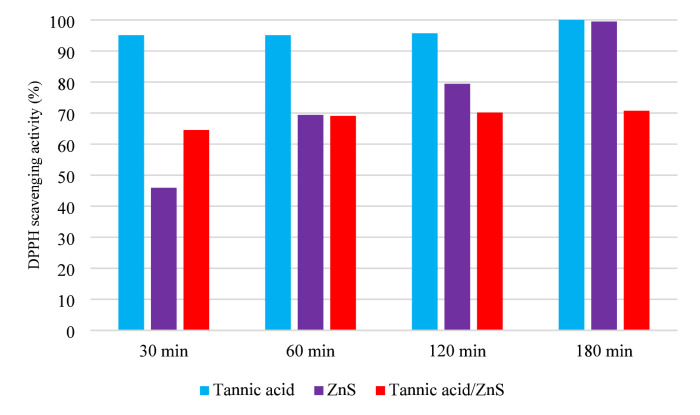
Antioxidant activity of tannic acid, ZnS, and TA/ZnS nanostructures at different time intervals (30 min, 60 min, 120 min, 180 min, and 48 h) using the DPPH radical scavenging assay.

**The antioxidant activity** of the TA/ZnS nanostructures was evaluated using the DPPH radical scavenging assay (Fig. 3). Tannic acid (TA) alone exhibited rapid and complete radical scavenging, reaching nearly 100% inhibition at 180 min, owing to its rich polyphenolic structure and multiple hydroxyl groups that act as effective hydrogen and electron donors^47^.

In contrast, ZnS nanoparticles displayed a slower but steady increase in activity, starting from 45.9% at 30 min and rising to 99.5% at 180 min, consistent with previous reports highlighting their moderate ROS-scavenging ability and high oxidative resistance^48^.

Interestingly, the TA/ZnS hybrid nanostructures exhibited a delayed but consistent antioxidant response: scavenging rose from 64.5% at 30 min to 70.7% at 180 min, indicating a controlled and sustained antioxidant release. This trend suggests that the interaction between TA and ZnS enables stabilization and gradual availability of active phenolic sites, as ZnS helps maintain dispersion and structural integrity of TA ^49^.

However, the lower final scavenging compared to TA alone implies that the immobilization of TA on ZnS may partially hinder the accessibility of some active phenolic sites. Despite this, the hybrid’s performance is well-suited for food packaging applications, where gradual and prolonged antioxidant protection is more desirable than rapid reactivity, as it can delay oxidative spoilage and extend shelf life^50^.

### Film characterization

#### FT-IR analysis

**Fig. 4:**
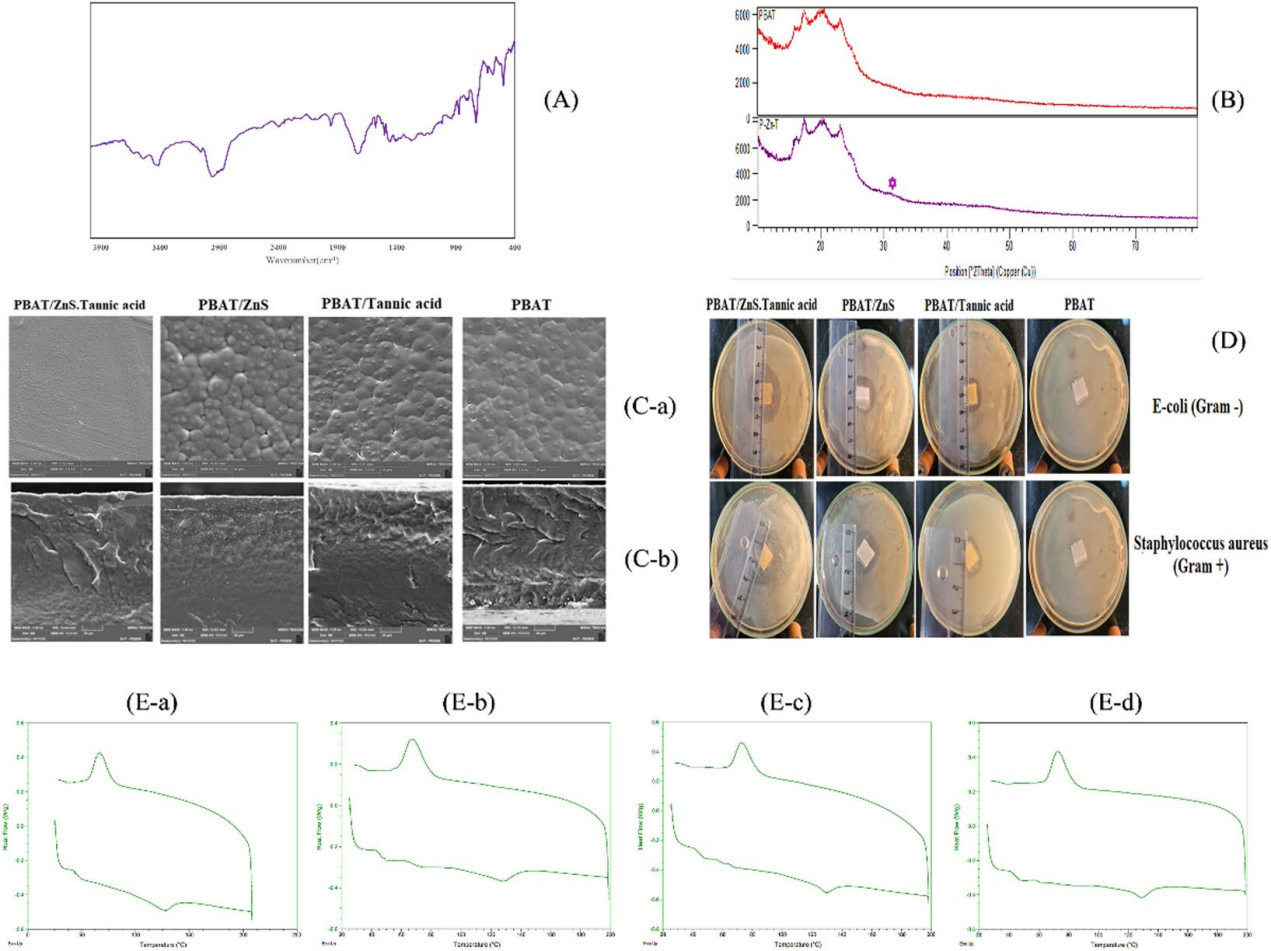
Characterization and antibacterial analysis of PBAT-based films; (**A**) FT-IR spectra showing functional groups of PBAT-based nanocomposites. (**B**) XRD curves of PBAT and PBAT/TA/ZnS. (C-a) Surface morphology and (C-b) cross-sectional FE-SEM images of PBAT-based films, including PBAT/TA/ZnS, PBAT/ZnS, PBAT/TA, and neat PBAT. (**D**) Antibacterial activity results against *E. coli* (Gram-negative) and *Staphylococcus aureus* (Gram-positive), demonstrating inhibition zones for PBAT/TA/ZnS, PBAT/ZnS, PBAT/TA, and neat PBAT. (**E**) DSC thermograms illustrating the thermal behavior of PBAT-based nanocomposite films, (E-a) PBAT, (E-b) PBAT/TA, and (E-c) PBAT/ZnS, and (E-d) PBAT/TA/ZnS.

The FT-IR spectroscopy was employed to examine the chemical structure and confirm the successful integration of TA/ZnS hybrid nanostructures into the PBAT matrix (Fig. 4(A)). The spectrum of the composite film exhibited a distinct absorption band at ~ 1700 cm⁻¹, attributed to the C = O stretching vibration of ester groups in PBAT. Another strong band at ~ 2930 cm⁻¹ was assigned to C–H stretching in the aliphatic segments of the polymer backbone^51^. Peaks at ~ 1167 cm⁻¹ and ~ 1269 cm⁻¹ further confirmed the presence of C–O stretching vibrations, typical of ester linkages.

The incorporation of tannic acid was evidenced by a broad band centered at ~ 3300 cm⁻¹, corresponding to the O–H stretching of phenolic groups, and a band at ~ 1400 cm⁻¹, linked to aromatic C = C vibrations. These features confirm the retention of tannic acid’s polyphenolic functionality, crucial for its antioxidant behavior^52,53^.

A weak but discernible absorption feature in the range of 500–600 cm⁻¹ was also observed, which is characteristic of Zn–S stretching vibrations^54,55^. This band confirms the formation and embedding of ZnS nanoparticles in the polymer matrix. Previous studies have reported Zn–S bands in this region in polymer-based and oxide-sulfide hybrid systems, supporting our spectral assignment^54,55^.

Taken together, the FT-IR data confirms the co-presence of PBAT, tannic acid, and ZnS nanostructures in the composite, with no evidence of major phase separation. The observed shifts and broadening suggest potential hydrogen bonding and interfacial interactions between the hybrid nanofillers and the PBAT matrix, which likely contribute to the film’s improved functional properties.

#### XRD analysis

The XRD patterns of the PBAT film and PBAT film containing TA/ZnS nanostructures are shown in Fig. 4(B). In the spectrum of the neat PBAT film, broad peaks observed at 2θ = 14.11° and 16.87° are characteristic of the semi-crystalline nature of PBAT^56^. These peaks can be attributed to the amorphous regions within the PBAT matrix, which typically display low crystallinity due to their polymeric chain structure. The broad nature of these peaks confirms the presence of disordered domains, which is consistent with the literature on PBAT.

In the XRD spectrum of the PBAT/TA/ZnS composite film, the characteristic peak of ZnS at 2θ = 31.8° becomes visible. This peak corresponds to the (200) crystal plane of the cubic ZnS structure, as referenced in the standard JCPDS card no. 05-0566 ^38^. The presence of this peak indicates the successful incorporation of ZnS nanostructures into the PBAT matrix. However, due to the dominant amorphous nature of PBAT, the crystalline peaks of ZnS are slightly masked, resulting in a lower intensity. This behavior is typical when inorganic nanoparticles are dispersed within semi-crystalline polymeric matrices^57,58^. Additionally, a weak peak at 2θ = 25° confirms the presence of tannic acid within the composite film. This peak aligns with the amorphous characteristics of tannic acid, which do not exhibit sharp crystalline reflections. The coexistence of ZnS and tannic acid within the PBAT matrix suggests uniform dispersion of the nanostructures without significant phase separation.

Overall, the XRD analysis demonstrates that the incorporation of TA/ZnS nanostructures does not significantly disrupt the semi-crystalline nature of PBAT. The appearance of the characteristic ZnS peak and the amorphous peak of tannic acid validates the formation of a composite film with retained structural integrity. The broadening of the peaks also suggests a nanoscale dispersion of the ZnS particles, i.e., essential for enhancing the film’s mechanical and barrier properties. This uniform incorporation contributes to the observed improvements in antioxidant activity, UV-blocking capability, and mechanical performance in subsequent analyses.

#### FE-SEM analysis

The surface and cross-sectional morphologies of the PBAT-based films were examined using field-emission scanning electron microscopy (FE-SEM), and the micrographs are shown in Fig. 4(C-a, b). The neat PBAT film exhibited a smooth, compact, and homogeneous surface, which is characteristic of the flexible and semi-crystalline nature of PBAT. The PBAT/TA film revealed a slightly rougher surface due to the partial agglomeration of tannic acid and its moderate interaction with the polymer chains. In contrast, the PBAT/ZnS film displayed noticeable surface roughness with visible nanoparticle clusters, indicating incomplete dispersion and mild aggregation of ZnS nanoparticles within the matrix.

Interestingly, the PBAT/TA/ZnS composite film showed a comparatively smoother and more uniform surface than the single-filler systems, suggesting enhanced compatibility and interfacial interaction between the hybrid nanostructures and the PBAT matrix. This synergistic effect likely results from the stabilizing action of TA on ZnS, improving its distribution within the polymer network^59^.

The cross-sectional images provided further insights into the internal structure of the films. The neat PBAT film displayed a layered and loosely packed morphology with observable voids, typical of polymers with low intermolecular interactions. The incorporation of TA or ZnS slightly enhanced the packing density, reducing voids and increasing structural integrity. Notably, the PBAT/TA/ZnS film exhibited the most compact and dense cross-section, with minimal phase separation or voids, indicative of strong interfacial adhesion and effective nanofiller distribution^60^. This improved morphology is expected to contribute positively to the mechanical strength, barrier properties, and overall performance of the composite films in food packaging applications.

#### Mechanical properties analysis

The mechanical properties of PBAT-based films, as summarized in Table 1, reveal the influence of tannic acid (TA), zinc sulfide (ZnS), and TA/ZnS hybrid nanostructures on tensile strength (TS), elastic modulus (EM), and elongation at break (EB). The neat PBAT film exhibited excellent mechanical performance with a TS of 30.04 MPa, EM of 80.00 MPa, and EB of 353%, reflecting its inherent ductility and semi-crystalline structure suitable for flexible packaging applications^61^.

Upon incorporation of TA (PBAT/TA), a noticeable decline in mechanical properties was observed: TS dropped to 23.97 MPa, EM to 73.34 MPa, and EB to 285%. This reduction may be attributed to the agglomeration of tannic acid within the matrix, which disrupts the polymer network and weakens interfacial adhesion between the filler and polymer chains^62^.

In contrast, the incorporation of ZnS nanoparticles (PBAT/ZnS) led to an enhancement in stiffness and strength, with TS and EM increasing to 25.94 MPa and 86.67 MPa, respectively. This improvement is attributed to the rigid nature and good dispersion of ZnS nanocrystals, which act as reinforcement agents by effectively transferring applied stress^63^. However, EB slightly decreased to 290%, likely due to local stress concentration and reduced matrix mobility^64^.

Interestingly, the PBAT/TA/ZnS composite film showed a further reduction in mechanical performance (TS = 20.85 MPa, EM = 70.00 MPa, EB = 274%). This outcome suggests that while the hybrid system offers multifunctionality, it also introduces challenges in achieving homogeneous dispersion and optimal interfacial interactions^65^.

Nevertheless, the mechanical values remain within acceptable ranges for flexible packaging applications^66,67^. Although a ~ 30% reduction in tensile strength was observed in the PBAT/TA/ZnS film compared to neat PBAT, this trade-off is compensated by the enhanced bioactive functionalities (e.g., antioxidant and UV-blocking properties) imparted by the TA/ZnS nanostructures^68^. All mechanical measurements were performed in triplicate. Mean values are reported, and standard deviations were calculated but not shown for brevity.

**Table 1:** Mechanical properties of PBAT-based films, including thickness, elastic modulus (EM), elongation at break (EB), and tensile strength (TS). All values are expressed as mean ± standard deviation (*n* = 3).

Films	Thickness) mm)	EM (MPa)	EB (%)	TS (MPa)
**PBAT**	0.153 ± 0.005	80.00 ± 5.00	353 ± 6.00	30.04 ± 2.50
**PBAT/TA**	0.120 ± 0.018	73.33 ± 15.28	284.67 ± 5.77	23.97 ± 2.99
**PBAT/ZnS**	0.155 ± 0.034	86.67 ± 15.28	290.00 ± 9.17	25.94 ± 3.87
**PBAT/TA/ZnS**	0.167 ± 0.002	70.00 ± 17.32	274.33 ± 20.22	20.85 ± 1.30

#### UV blocking and transparency analysis

The UV-blocking and transparency properties of PBAT-based films were evaluated at two specific wavelengths: 330 nm for UV-blocking capability and 660 nm for film transparency.

At 330 nm (UV range), the neat PBAT film exhibited the highest transmittance of 41%, indicating its poor UV-blocking performance due to the absence of UV-absorbing functional groups and its inherently transparent nature. Incorporating tannic acid into the PBAT matrix (PBAT/TA) significantly improved UV-blocking efficiency, reducing the transmittance to 12%. This enhancement arises from the UV-absorbing phenolic groups in tannic acid, which effectively absorb and neutralize UV radiation. Addition of ZnS nanoparticles (PBAT/ZnS) further improved UV-blocking performance, with UV transmittance reduction down to 28%. This behavior is attributed to the semiconductor properties of ZnS nanoparticles and their ability to absorb UV radiation due to their wide bandgap (~ 3.7 eV (^69^. Notably, the PBAT/TA/ZnS composite film demonstrated the most effective UV-blocking capability, with UV transmittance dropping to 3%. This remarkable improvement results from the synergistic effect of ZnS nanoparticles and tannic acid, where ZnS provides strong UV absorption and tannic acid enhances this effect through its polyphenolic structure, creating a highly efficient UV barrier.

At 660 nm (visible range), corresponding to film transparency, the neat PBAT film displayed the highest transmittance of 44.4%, confirming its excellent optical clarity due to its homogeneous structure and lack of nanofiller incorporation. In contrast, the PBAT/ZnS and PBAT/TA films showed reduced transparency, with transmittance values of 25.5% and 25.5%, respectively. This reduction can be attributed to light scattering caused by the dispersed ZnS nanoparticles and tannic acid aggregates within the polymer matrix. The PBAT/TA/ZnS composite film exhibited the lowest transmittance of 13.2%, indicating the greatest reduction in transparency. This effect results from the combined scattering and absorption caused by the hybrid nanostructures, which disrupt the uniformity of the PBAT matrix.

While the reduction in transparency at 660 nm represents a trade-off, the substantial improvement in UV-blocking properties at 330 nm outweighs this limitation for applications where UV protection is prioritized over optical clarity. The PBAT/TA/ZnS composite film demonstrates superior UV shielding (3% transmittance) and a well-balanced structural performance, i.e., an ideal candidate for active food packaging applications where UV protection is critical to preventing photodegradation and extending shelf life.

#### Water vapor permeability (WVP) and water contact angle (WCA) analysis

The water vapor permeability (WVP) and water contact angle (WCA) of PBAT-based films were evaluated to determine their barrier properties and surface hydrophobicity, both of which are critical for food packaging applications. (Table 2)

##### Water vapor permeability (WVP)

The neat PBAT film exhibited the highest WVP value of 6.38 × 10⁻¹^2^ g.m/m².s. Pa, reflecting its relatively low barrier performance against moisture due to its semi-crystalline structure, which allows water vapor diffusion. Incorporation of tannic acid into PBAT (PBAT/TA) significantly reduced the WVP to 3.12 × 10⁻¹^2^ g.m/m².s.Pa. This improvement results from the crosslinking interactions of tannic acid, as confirmed by FT-IR analysis. The C = O stretching band of PBAT shifted from 1712 → 1705 cm⁻¹ and the broad O–H band from 3300 → 3270 cm⁻¹ upon incorporation of tannic acid and TA/ZnS nanostructures, evidencing hydrogen bonding and partial ester–phenol interactions between TA and PBAT. These interfacial interactions reduce the polymer chain mobility and free volume, thereby restricting water vapor diffusion and improving the overall barrier performance. The PBAT/ZnS film further lowered the WVP to 4.27 × 10⁻¹^2^ g.m/m².s.Pa, as ZnS nanoparticles act as impermeable fillers, creating a “tortuous path” for vapor transmission and effectively increasing the diffusion distance. Notably, the PBAT/TA/ZnS film achieved the lowest WVP value of 2.77 × 10⁻¹^2^ g.m/m².s.Pa, demonstrating a synergistic effect of ZnS nanoparticles and tannic acid. ZnS nanoparticles provide physical barriers, while tannic acid reinforces the matrix via crosslinking, leading to a compact and impermeable structure. These results confirm the suitability of the hybrid nanostructure for applications where moisture control is critical to preserving food quality and extending shelf life^70^.

##### Water contact angle (WCA)

The neat PBAT film exhibited a WCA of 72.2°, indicating moderate hydrophilicity due to the presence of polar groups in its molecular structure, which reduces surface hydrophobicity. Incorporation of tannic acid increased the WCA to 78.2°, suggesting a slight improvement in hydrophobicity. This is attributed to tannic acid’s aromatic structure and hydroxyl groups, which reduce surface wettability. Conversely, incorporating ZnS nanoparticles (PBAT/ZnS) resulted in a lower WCA of 65.0°, indicating increased hydrophilicity. This decrease is primarily attributed to the exposure of polar Zn–S sites, which enhance surface energy and water affinity, despite a slight increase in roughness.

The PBAT/TA/ZnS film showed the highest WCA value of 81.6°, representing a significant improvement in hydrophobicity. This enhancement arises from the combined effects of surface chemistry and morphology. The presence of tannic acid introduces aromatic and less polar functional groups that lower surface energy, while ZnS nanoparticles contribute to moderate surface roughness. According to the Cassie–Baxter model, this hierarchical surface texture amplifies the intrinsic hydrophobic behavior, resulting in improved water repellency and reduced moisture absorption.

Overall, the integration of ZnS nanoparticles, tannic acid, and their hybrid nanostructures into PBAT films significantly improves both water vapor barrier performance and surface hydrophobicity. The PBAT/TA/ZnS composite film exhibits the most balanced and superior performance, achieving the lowest WVP and the highest WCA. These enhancements highlight the potential of these nanocomposite films for advanced food packaging applications, where moisture resistance and surface properties are essential for maintaining food quality and extending shelf life. Barrier and surface property measurements were also conducted in triplicate. Only mean values are presented in the main text.

**Table 2:** Water vapor permeability (WVP) and water contact angle (WCA) of PBAT-based films, including thickness measurements. All values are expressed as mean ± standard deviation (*n* = 3).

Films	Thickness (mm)	WVP (×10^− 12^ g.m/m^2^.Pa.s)	WCA (deg.)
**PBAT**	0.153 ± 0.005	6.38 ± 0.22	72.2 ± 1.5
**PBAT/TA**	0.120 ± 0.018	3.12 ± 0.18	78.2 ± 1.2
**PBAT/ZnS**	0.155 ± 0.034	4.27 ± 0.20	65.0 ± 1.8
**PBAT/TA/ZnS**	0.167 ± 0.002	2.77 ± 0.15	81.6 ± 1.4

#### Differential scanning calorimetry (DSC) analysis

The DSC thermograms of PBAT-based films, including pure PBAT (Fig. 4(E-a)), PBAT/TA (Fig. 4(E-b)), PBAT/ZnS (Fig. 4(E-c)), and PBAT/TA/ZnS (Fig. 4(E-d)), provide valuable insights into their melting transitions and crystalline behavior.

The DSC thermogram of the pure PBAT (Fig. 4(E-a)) displays a well-defined melting peak, characteristic of its semi-crystalline nature, with the melting temperature (Tm) observed in the range of 120–130 °C. This result aligns with literature values for neat PBAT. A minor peak around 50 °C, attributed to surface contamination (e.g., skin oils during handling), is negligible and does not impact the overall thermal analysis.

The addition of tannic acid (PBAT/TA) did not significantly alter the Tm, indicating that the crystalline structure of PBAT was largely preserved (Fig. 4(E-b)). This observation suggests that tannic acid was well-dispersed within the polymer matrix without interrupting PBAT chain packing.

The DSC thermogram of the PBAT/ZnS film (Fig. 4(E-c)) similarly shows a melting temperature in the range of 120–130 °C, comparable to that of neat PBAT. The minimal change in melting behavior implies that ZnS acts as an inert filler with limited influence on PBAT crystallization.

For the PBAT/TA/ZnS composite film (Fig. 4(E-d)), no significant shift in Tm is observed relative to neat PBAT. The DSC thermogram of PBAT/TA/ZnS also displayed a single melting peak near 122 °C, with no additional transitions. This consistency indicates that the TA/ZnS hybrid nanostructures are well-integrated into the PBAT matrix and do not induce phase separation or new crystalline domains.

Overall, the near-constant melting temperature across all formulations indicates that the fillers did not significantly modify PBAT crystallinity. However, it should be noted that the invariance in Tm does not directly reflect thermal stability, which was instead evaluated by TGA analysis. The DSC results mainly confirm that the polymer’s crystalline structure remains intact upon incorporation of TA, ZnS, and TA/ZnS, supporting good miscibility and dispersion of the fillers.

#### Antioxidant activity analysis

**Fig. 5:**
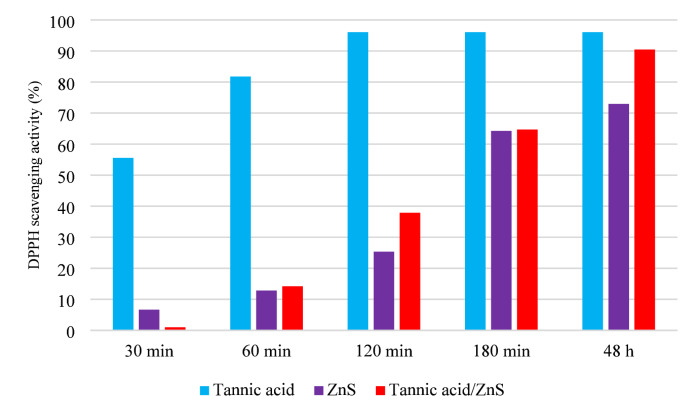
Antioxidant activity of PBAT-based films with tannic acid, ZnS, and TA/ZnS nanostructures at different time intervals (30 min, 60 min, 120 min, 180 min, and 48 h) using the DPPH radical scavenging assay.

The antioxidant activity of PBAT-based films containing tannic acid, ZnS, and their hybrid nanostructures was evaluated over various time intervals (30 min to 48 h) using the DPPH radical scavenging assay, as shown in Fig. 5. Pure tannic acid films exhibited the highest and most rapid antioxidant response, with activity increasing from approximately 55–60% at 30 min to nearly 100% at 48 h. This strong performance is attributed to the high density of phenolic hydroxyl groups capable of efficient radical scavenging.

ZnS nanoparticles alone demonstrated a relatively low initial activity (< 10% at 30 min), but their performance improved gradually to ~ 60% by 48 h, suggesting limited direct antioxidant capacity but some oxidative stability over time.

Notably, the PBAT/TA/ZnS composite film showed very low initial scavenging activity (< 10% at 30 min), which gradually increased to approximately 90% at 48 h. This slow but steady progression suggests a controlled release mechanism, where the surface interactions between ZnS and tannic acid molecules modulate the availability of active phenolic groups over time. The uniform dispersion of the hybrid nanostructure within the PBAT matrix likely contributes to this sustained antioxidant profile.

This sustained release is particularly advantageous for active food packaging applications, where long-term antioxidant protection is required to inhibit lipid oxidation and delay spoilage. Compared to free tannic acid, which provides a burst-release effect, the hybrid nanostructure offers prolonged functionality, while the PBAT matrix further facilitates gradual diffusion and stability of the active components.

Overall, the antioxidant results highlight the functional synergy of the TA/ZnS hybrid system and its effective integration into PBAT films, offering a promising platform for extended antioxidant protection in real-world packaging scenarios. Future studies could further investigate the release kinetics of tannic acid from the hybrid structures to fine-tune the antioxidant delivery profile.

#### Antibacterial activity analysis

The antibacterial activity of PBAT-based films was evaluated using the zone of inhibition method, where the clear zone surrounding each film sample indicates the inhibition of bacterial growth. The results, presented in Fig. 4(D), highlight the performance of different nanocomposite films against Gram-negative *E. coli* and Gram-positive *Staphylococcus aureus* bacteria.

The pristine PBAT film (control) exhibited no antibacterial activity, as evidenced by the complete bacterial growth around the film. This result confirms the lack of inherent antibacterial properties in pure PBAT. In contrast, the PBAT/TA film displayed moderate antibacterial activity, with inhibition zones of 2.00 cm for *E. coli* and 2.01 cm for *S. aureus*. The antibacterial efficacy of tannic acid arises from its polyphenolic structure, which disrupts bacterial cell membranes by interacting with proteins and lipid bilayers, leading to cell damage^71–73^.

The PBAT/ZnS film demonstrated improved antibacterial activity, producing inhibition zones of 2.53 cm for *E. coli* and 2.41 cm for *S. aureus*. The enhanced antibacterial activity of the PBAT/ZnS film can be attributed to the photocatalytic nature of ZnS nanoparticles, which are known to produce ROS under light exposure, causing oxidative damage to bacterial membranes and intracellular components, as reported in previous studies^74,75^.

The most significant antibacterial performance was observed in the PBAT/TA/ZnS film, which achieved inhibition zones of 3.01 cm for *E. coli* and 3.04 cm for *S. aureus*. This synergistic effect results from the combined mechanisms of tannic acid and ZnS nanostructures: tannic acid disrupts bacterial membranes, while ZnS generates ROS, amplifying the antimicrobial action. The uniform dispersion of TA/ZnS nanostructures within the PBAT matrix further optimizes their activity by increasing the contact area with the bacterial environment.

The results demonstrate a synergistic improvement in antibacterial performance when tannic acid and ZnS are combined, outperforming their contributions. The films effectively inhibit both Gram-negative and Gram-positive bacteria, highlighting their versatility for broad-spectrum antimicrobial applications. These findings underscore the potential of PBAT/TA/ZnS nanocomposite films for active food packaging, where bacterial contamination poses a significant challenge. The superior antibacterial properties of these films make them ideal candidates for extending the shelf life of perishable food products while ensuring food safety.

## Conclusion

This study demonstrates the successful fabrication of a multifunctional and biodegradable film based on poly(butylene adipate-co-terephthalate) (PBAT), reinforced with in situ synthesized tannic acid/zinc sulfide (TA/ZnS) hybrid nanostructures. The integration strategy facilitated nanoscale synergy between the organic (polyphenolic TA) and inorganic (ZnS) components, yielding enhanced functional performance.

The resulting films exhibited sustained antioxidant activity (90.46% DPPH scavenging after 48 h), indicating a delayed yet prolonged release profile suitable for long-term oxidative protection in active food packaging. Strong antibacterial activity (inhibition zones > 3.0 cm) and exceptional UV-shielding capacity (up to ~ 97%) were also achieved.

Structural and morphological analyses confirmed the successful in situ incorporation and uniform dispersion of hybrid fillers within the PBAT matrix, without disrupting its crystallinity or thermal stability. Moreover, the hybrid films displayed improved moisture barrier performance (40.9% reduction in WVP) and enhanced surface hydrophobicity (13% increase in contact angle), essential for packaging efficiency.

Nevertheless, a moderate reduction in mechanical performance was observed upon incorporation of the hybrid nanostructures, particularly in tensile strength (approximately 30% decrease) and elongation at break. This decline is likely due to the challenges associated with homogeneous dispersion and interfacial compatibility. Despite this, the films maintained sufficient ductility and mechanical integrity for flexible packaging use. Future optimization —such as adjusting filler loading or introducing compatibilizers—could help mitigate this limitation while preserving the film’s multifunctional benefits.

In conclusion, this study highlights a green and scalable in situ synthesis strategy to fabricate bioactive polymeric films with multifunctional performance. The combination of biodegradability, enhanced protective properties, and industrial feasibility positions TA/ZnS-based nanocomposite films as promising candidates for next-generation sustainable food packaging. Further investigations should focus on process upscaling, food–film interaction studies, and long-term stability under real storage conditions.

## Data Availability

The datasets generated and/or analysed during the current study are available from the corresponding author on reasonable request.
